# Infective Endocarditis in the Elderly: Challenges and Strategies

**DOI:** 10.3390/jcdd9060192

**Published:** 2022-06-17

**Authors:** Carlos Bea, Sara Vela, Sergio García-Blas, Jose-Angel Perez-Rivera, Pablo Díez-Villanueva, Ana Isabel de Gracia, Eladio Fuertes, Maria Rosa Oltra, Ana Ferrer, Andreu Belmonte, Enrique Santas, Mauricio Pellicer, Javier Colomina, Alberto Doménech, Vicente Bodi, Maria José Forner, Francisco Javier Chorro, Clara Bonanad

**Affiliations:** 1 Servicio de Medicina Interna, Hospital Clínico Universitario de Valencia, 46010 Valencia, Spain; carlos.bea@outlook.com (C.B.); sara.vela.b@gmail.com (S.V.); anadegrale@gmail.com (A.I.d.G.); eladio.fuertes@hotmail.com (E.F.); mrosaoltra@gmail.com (M.R.O.); ferrerllusar@hotmail.com (A.F.); anbeldo@hotmail.com (A.B.); maria.jose.forner@uv.es (M.J.F.); 2 Servicio de Cardiología, Hospital Clínico Universitario de Valencia, 46010 Valencia, Spain; sergiogarciablas@gmail.com (S.G.-B.); ensantas@gmail.com (E.S.); mauriciopellicer@gmail.com (M.P.); vicente.bodi@uv.es (V.B.); francisco.j.chorro@uv.es (F.J.C.); 3 Instituto de Investigación Sanitaria INCLIVA, 46010 Valencia, Spain; 4 Servicio de Cardiología, Hospital Universitario de Burgos, 09003 Burgos, Spain; jangel.perezrivera@gmail.com; 5 Servicio de Cardiología, Hospital Universitario La Princesa, 28006 Madrid, Spain; pablo_diez_villanueva@hotmail.com; 6 Servicio de Microbiología, Hospital Clínico Universitario de Valencia, 46010 Valencia, Spain; jcolominarodri@yahoo.es; 7 Servicio de Cirugía Cardiovascular, Hospital Clínico Universitario de Valencia, 46010 Valencia, Spain; domenech46@gmail.com; 8 Departamento de Medicina, Universidad de Valencia, 46010 Valencia, Spain; 9 Centro de Investigación Biomédica en Red-Cardiovascular, 28029 Madrid, Spain

**Keywords:** infective endocarditis, elderly, diagnosis, treatment

## Abstract

The specific management of infective endocarditis (IE) in elderly patients is not specifically addressed in recent guidelines despite its increasing incidence and high mortality in this population. The term “elderly” corresponds to different ages in the literature, but it is defined by considerable comorbidity and heterogeneity. Cancer incidence, specifically colorectal cancer, is increased in older patients with IE and impacts its outcome. Diagnosis of IE in elderly patients is challenging due to the atypical presentation of the disease and the lower performance of imaging studies. Enterococcal etiology is more frequent than in younger patients. Antibiotic treatment should prioritize diminishing adverse effects and drug interactions while maintaining the best efficacy, as surgical treatment is less commonly performed in this population due to the high surgical risk. The global assessment of elderly patients with IE, with particular attention to frailty and geriatric profiles, should be performed by multidisciplinary teams to improve disease management in this population.

## 1. Introduction

Infective endocarditis (IE) is still associated with high mortality and morbidity despite significant advances in recent decades [1,2,3,4]. Although it is considered an uncommon disease, the incidence of IE is especially high in patients above 70 years of age [4], and it is expected to increase in the following years due to the growing life expectancy [5] and the medicalization of global health [6,7,8]. Peculiarities of IE in the elderly are not specifically addressed in current guidelines [9,10] despite the higher mortality and functional impact of the disease in this population [11,12,13]. Furthermore, clinical presentation, diagnosis, and management differ from those in younger patients [14,15,16,17]. This review summarizes the challenges and uncertainty areas of IE management in elderly patients, trying to provide strategies to overcome them.

## 2. Epidemiology: A Growing Problem

The estimated annual incidence of IE is 3 to 15 cases per 100,000 [1,18,19,20,21,22], with wide regional variations. Despite the decline of rheumatic heart disease, a formerly widespread predisposing condition [23], the incidence appears to be increasing, especially at the expense of the elderly [24]. There is debate about the last restriction for antibiotic prophylaxis in international guidelines in this trend [25,26]. Still, other epidemiological changes from the last decades with enough influence need to be addressed [1,24,27].

First, life expectancy is increasing in high-income countries, nowadays being more than 80 years [5]. Accordingly, in Western countries, patients with IE are significantly older, with up to one-third of patients older than 70 years old [15,24,28]. Indeed, age itself is a risk factor, as older patients are estimated to have a five-fold higher risk of endocarditis [4,29]. 

Additionally, multiple studies addressing specific characteristics of IE in the elderly have found a high rate of comorbidities in this population (Table 1) [12,16,17,30,31,32,33]. However, due to the lack of large multicentric cohorts [34] and the heterogeneity of this population, there is not enough evidence to support specific recommendations, although some have made attempts [15,27].

In this regard, not even the term “elderly” is equally applied in the literature, having been used by international guidelines for patients older than sixty [9,10], but more recently for older than eighty. Meanwhile, some preferred to use the term “very elderly” [17] for over eighty-year-old patients, while others have referred to the term “octogenarian” [30]. In any case, aging is not a uniform process, including many different profiles [27].

Some authors have tried to define these profiles and compared them between different age groups. For example, Oliver et al. show that patients over 80 years old have more comorbidities, among the most common being cancer, diabetes, and renal disease, along with higher Charlson index and EuroSCORE, compared with younger patients [17,30]. Chronic heart disease and high blood pressure also have a higher prevalence [12,16,17,32,33]. Compared to the predominance of male sex in IE incidence among younger patients [31,34], some studies have found similar proportions of men and women among octogenarians, which could be partially attributable to women’s higher life expectancy [17]. 

Due to cardiac morbidities and aging, the prevalence of cardiovascular implantable devices and prosthetic valves is higher, including transcatheter aortic valve implantation (TAVI), which are well-known risk factors for IE, with even worse prognosis [19,31]. Moreover, predisposing events such as surgery, instrumentation, and recurrent infections are more common, being at high risk for bloodstream invasion [31,35]. This frequent contact with health care implies a higher rate of nosocomial infections [15] and resistant pathogens [32]. 

The risk of hematological and abdominal cancer increases in the first three months after IE diagnosis and remains higher than expected for up to 12 months in the case of abdominal cancer [36]. In addition, the incidence of IE in the elderly (and not other infections) increases up to fivefold around the diagnosis of colorectal cancer (CRC) compared with the diagnosis of lung, breast, or prostate cancer, or an arbitrary date for individuals without cancer [37]. CRC (or preneoplastic lesions such as adenomas) can cause IE by allowing the translocation of bacteria [38]. Still, there are other possible explanations for this association: IE patients often receive a colonoscopy as a part of the diagnostic work-up (indicated routinely in the case of *S. gallolyticus* IE [9,39,40,41] and increasingly recommended in *E. faecalis* IE [42,43,44,45,46]), leading to higher CRC detection; colonoscopy could increase itself the risk of IE by disrupting the bowel mucosa and allowing translocation; they share common etiologic factors. Concomitant diagnosis increases non-cancer-specific mortality and can produce changes in cancer treatment based on the predicted survival of patients [37].

Comorbidities could play not only a pathogenic role [4,32] but also have a strong influence on the outcome as in treatment selection [47]. Some studies have shown this population to have higher 1-year mortality than younger cohorts, although not higher global mortality [30]. Despite the recommendations of international guidelines, age has been found to be the main factor involved in the restrictive use of surgery [47,48]. Non-performance of surgery [29,30], along with the Charlson Comorbidity index and nutritional and functional status, was independently associated with mortality [12].

## 3. Clinical Presentation: The Impact of Comorbidities and Nonspecificity

**Figure 1:**
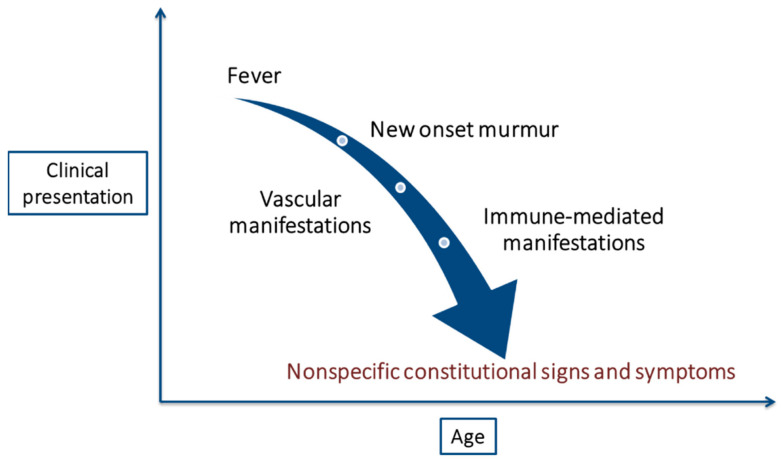
Changes in the clinical presentation of infective endocarditis with age.

More than a century after the first description of IE, this disease remains notable for its diverse and nonspecific presentation (Table 2), which often leads to a late diagnosis [49]. Furthermore, the clinical presentation of this disease in elderly patients is considered atypical [17], with nonspecific constitutional signs and symptoms, such as lethargy, fatigue, malaise, anorexia, and weight loss, being the most frequent manifestations. Clinical presentation as delirium is characteristic in this age group and can sometimes be the only manifestation [4]. However, fever, the most common initial symptom in the general population, is frequently absent in elderly patients [14]. Additionally, the high prevalence of baseline murmurs in older adults makes this finding nonspecific unless the murmur is clearly new [49]. Both the absence of fever and new-onset heart murmurs contribute to the delayed diagnosis of IE [31] (Figure 1).

Classic IE stigmas, surprisingly rare in the general population [49], are even less frequent in the elderly. Despite higher rates of mitral valve involvement, vascular manifestations of IE, such as embolic events, are less common in the elderly [14]. It could be explained by the widespread use of antiplatelets and anticoagulants among the older population. Immune-mediated manifestations are also less frequent, probably due to a less intense acute immune response. These two factors together (higher antiplatelet/anticoagulant use and weaker immune response) may help to explain the lower rate of vegetations found in this population group [32].

Therefore, we are faced with a vulnerable population group, in which the diagnosis is often more difficult due to the nonspecificity of its presentation and the higher prevalence of comorbidities compared to the general population [4].

## 4. Microbiology: From *S. viridans* to Enterococcal Etiology. Adequate Sample Collection Saves Lives

As previously stated, the diagnosis of IE in the elderly is rarely straightforward and is often delayed or overlooked because of atypical clinical presentation. Therefore, with clinical manifestations and imaging tests, the confirmatory diagnosis of IE must be based on microbiological findings [10].

Gram-positive cocci represent 80–90% of IE cases. The main microorganism isolated in young adults is *Staphylococcus aureus*, followed by the *Streptococcus viridans* group (includes *S. anginosus*, *S. bovis/equinus*, *S. mitis*, *S. sanguinis*, *S. mutans*, and *S. salivarius*, all commensals of the oral cavity and gastrointestinal tract) and *Enterococcus* spp. However, in patients 65 to 79 years old, *Enterococcus* spp. is detected more frequently. In octogenarians, *S. viridans* group and *Enterococcus* spp. are mainly isolated, with a few cases due to *S. aureus* (usually resistant to methicillin because of frequent exposure to medical attention). The isolation of coagulase-negative staphylococci is equally frequent in all age groups, although somewhat more frequent in patients older than 65 years and carriers of prosthetic devices or valves [17]. *Streptococcus gallolyticus* (formerly *S. bovis*) is noted for causing IE associated with underlying colonic pathology (ulcers, diverticular disease, and malignancy), which provides a gateway for bacteremia [39,40]. The association between *E. faecalis* IE and colonic pathology has also been described in the past few years [41,42,43,44,46].

Non-HACEK (*Haemophilus*, *Aggregatibacter*, *Cardiobacterium*, *Eikenella*, and *Kingella*) Gram-negative bacilli (GNB) constitute a rare cause of IE that has become more frequent than HACEK in recent years [51] due to the increase in age and comorbidity in the general population and the increase in enterobacterial bacteremia incidence [52,53,54]. Non-HACEK GNB Enterobacterales cases, predominantly caused by *E. coli* and more commonly associated with community acquisition, mitral valve involvement, genitourinary origin, and septic shock, were more frequent than non-HACEK GNB IE by non-fermentative GNB, principally *P. aeruginosa*, which is characterized by right valve involvement and health care association, predominantly venous catheter-related infection [51,55,56,57].

The cornerstone of the microbiological diagnosis of IE in all age groups continues to be blood culture (BC). Regardless of presentation, patients with unexplained persistent bacteremia or an unknown focus should be studied. The responsible organism can be recovered in 85–90% of patients by obtaining three blood samples collected at intervals of at least 30–60 min. It is not essential to perform the extraction coinciding with fever peaks since bacteremia in IE is constant. In positive blood cultures, the isolated microorganisms must be identified using highly reliable techniques (e.g., MALDI-TOF), as well as an antibiogram with determination of the MIC. Blood extractions through catheters should be avoided due to the difficulty of interpretation in cases of isolation of coagulase-negative staphylococci [9,10].

Approximately 10–15% of patients with IE have negative BC [58], making diagnosis even more complex and thus worsening the prognosis. Despite a lower risk clinical profile, these patients present higher in-hospital mortality than those with known etiology due to delayed diagnosis and, consequently, late initiation of antibiotic treatment [59]. The most common cause is the use of antibiotics before acquiring blood cultures (it is recommended to use blood culture bottles containing antimicrobial inhibitor resins). However, other possible explanations include infection by fastidious bacteria or fungi or alternative diagnoses such as non-bacterial thrombotic endocarditis (associated with hypercoagulable states or advanced neoplasms). Additional microbiological testing can identify a responsible organism in approximately two-thirds of patients. Thus, if blood cultures are negative at 5–7 days, serological tests for atypical organisms (*Bartonella*, *Brucella*, *Coxiella*, *Chlamydia*, *Legionella*, and *Mycoplasma*) should be considered [10,60].

Molecular techniques play a complementary role if embolic or valve material is available. The universal broad-spectrum polymerase chain reaction (PCR) for detecting 16s (bacteria) or 18S (fungi) rRNA is a sensitive technique that amplifies small amounts of microbial DNA and can identify the specific organism if combined with sequencing. This is particularly valuable in patients with previous exposure to antibiotics (since bacterial DNA often persists) and for non-culturable pathogens such as *Tropheryma whipplei*. However, false-positive results can arise due to sample contamination and PCR can also remain positive after eradicating viable bacteria (and, therefore, should not be used to guide the duration of therapy). New techniques that combine PCR with mass spectrometry hold promise for the direct characterization of bacteria in valve tissue [61].

## 5. Imaging Studies: Underperformance and Previous Valve Degeneration

Cardiac imaging techniques are one of the cornerstones in the diagnosis of IE. Echocardiography stands as the first line for the diagnosis and management of these patients [62]. Specifically, transesophageal echocardiography (TEE) provides additional value due to its higher resolution the better visualization of valvular structures. Other imaging techniques, such as CT, CMR, or PET, play a complementary role in specific cases [63].

Transthoracic echocardiography (TTE) must be performed promptly when IE is suspected. If TTE is suggestive of IE, TEE is indicated to better assess the extension of the infection and the valvular function. A TEE must also be performed in case of high clinical suspicion of IE if TEE is negative or non-diagnostic or in the case of prosthetic valves. Moreover, repeated TTE and/or TEE should be made 5–7 days after initial imaging if clinical suspicion of IE remains high [9,62,63]. Major diagnostic criteria of IE in echocardiography are vegetations, abscess or pseudoaneurysm, new valve regurgitation, and new dehiscence of a prosthetic valve. The characterization of these findings is also relevant for prognosis assessment and to guide therapeutic decisions. Thus, large vegetations, periannular complications, or severe valvular regurgitation pose a higher risk and support surgical indications in many cases. Three-dimensional TEE may provide additional value for this purpose, as it can better characterize vegetation size and perivalvular extension [64]. Follow-up echocardiography is recommended if a change in clinical condition occurs after initial diagnosis and to monitor response to medical therapy.

These recommendations apply to older patients, but some differences may be noted. López-Wolf et al. analyzed a cohort of 582 patients diagnosed with possible or definitive IE, comparing clinical and echocardiographic findings in three age groups: <65, 65–79, and ≥80 years [31]. TEE showed higher performance than TTE regardless of the age group. Vegetations were less likely to be detected in octogenarian patients than in their younger counterparts, both with TTE and TEE. TTE underperformance in the elderly may be related to a suboptimal acoustic window, whereas TEE can be attributed to degenerative changes in valvular structure. Valve degeneration, including fibrosis and calcification, is widespread in older patients, and it can be challenging to identify vegetation, even with TEE [31,34].

Multislice computed tomography (MSCT) provides high resolution to detect and characterize the perivalvular extension of IE (abscess, pseudoaneurysm, fistula) [65]. This technique may also be helpful in the case of prosthetic valve infection [66]. CT can diagnose IE complications such as embolization and its complications (infarcts, abscesses, etc.) [9]. It may also be used for coronary assessment before surgery in cases with a low risk of coronary artery disease [67]. MRI has a higher sensitivity than CT for detecting cerebral embolism [68]. Nuclear imaging techniques, such as SPECT and PET, are useful in borderline cases (i.e., possible IE using the Duke criteria), mainly if prosthetic material is affected [69]. 

## 6. Global Assessment of the Elderly Patient with IE: Well Worth the Effort

Since antibiotic prophylaxis when indicated and other preventive measures are often not enough to prevent IE in elderly people, we should make an effort to improve its management to decrease its burden on the older population.

Age appears as a major risk factor for death regardless of concomitant comorbidities, but there is no clear explanation for this observation [32,34,70,71]. Aging is a heterogeneous process that gives rise to a wide variety of patient profiles ranging from healthy aging to bedridden patients. There is a continuum of frail patients with diverse functional abilities, nutritional status, cognition, and comorbidities, which explains the clinical variability between patients of the same chronological age.

Usual health conditions in frail individuals, such as cognitive impairment, delirium, falls, or urinary incontinence, can be characterized by a comprehensive geriatric assessment (CGA) [72]. The CGA includes a team consisting of geriatric specialists, nurses, and social workers that provides information not only on the clinical, functional, and cognitive status of patients but also assesses polypharmacy, goals of care, and living will. CGAs are increasingly applied to specific conditions, such as infective endocarditis [73]; choosing between surgery or transcatheter aortic valve replacement (TAVR) for patients with aortic stenosis [74]; vascular surgery [75]; and postoperative mortality risk [76].

The Elderly IE study by Forestier et al. reveals that IE dramatically impairs the functional status of the oldest patients. The functional and nutritional status were the only parameters associated with mortality independently of cardiac and infectious characteristics of IE. In this cohort, despite improving mobilization, nutrition, and treatment by the geriatrician, around 10% still required physical restraint because of agitation, fell, or developed pressure ulcers. These results show that the management of older patients with IE includes not only antibiotics and surgical decisions but also preventing and treating recurrent complications in the older population, such as delirium, malnutrition, functional decline, and adverse drug effects [73].

In the K.J. Lu et al. retrospective observational study, Charlson Co-morbidity Index > 3 was a strong predictor of mortality, in addition to age, new heart failure, and anemia over long-term follow-up in infective endocarditis [13].

In Rodriguez Villarreal et al.’s cohort, Fried’s criteria (unintentional weight loss, exhaustion, low physical activity, slowness, and weakness) have recently been evaluated in elderly patients with advanced chronic kidney disease. The recognition of frailty in old-age patients could be useful in order to provide information and make decisions regarding treatment options [77,78,79].

Likewise, Olga H. Torres et al. highlight the importance of functional assessment of elderly patients with community-acquired pneumonia in hospitals. A basic evaluation of the activity of daily living in the emergency department could provide information about mortality risk and would help physicians in clinical decision-making without overestimating risk related to chronological age [80].

“Endocarditis teams” have been created in many reference centers, and studies have shown their impact, with improvements in early diagnosis, management strategies, and survival [13]. Incorporating geriatricians can help individualize treatment, placing management decisions in the context of risks, burdens, benefits, and prognosis, including remaining life expectancy, functional status, quality of life, and patient and family preferences and ethical aspects [81].

Since there is no specific management for elderly patients in the reference guidelines, we propose a comprehensive assessment with reliable instruments or scales validated to study functional status in older adults (Table 3).

## 7. Antibiotic Treatment: The Shorter, the Better. Diminishing Interactions and Adverse Effects

IE treatment requires high-dose and prolonged (2 to 6 weeks) antibiotic therapies regardless of the surgery’s indication and timing [4]. Although there is consensus among experts on the principles of IE antibiotic treatment, all recommendations in recent international guidelines are based on B-C evidence levels [9,10]. Moreover, specific recommendations for older patients are not included. Nevertheless, some aspects should be taken into account.

First, the plasmatic concentration of antibiotics can vary significantly with age due to altered renal and liver function [4], lower plasma albumin concentration (reduced transport of highly protein-bound drugs and their resulting free fraction) [15], fat mass index (changes in the volume of distribution alteration), etc. Thus, high antibiotic doses required in IE treatment imply an increased risk of adverse events (AEs) in the elderly: renal toxicity mainly caused by aminoglycosides and glycopeptides, but also by cloxacillin [93], neurological effects induced by high doses of penicillin, gastrointestinal adverse effects with rifampicin, etc. [94]. Although specific equations particularly developed to estimate glomerular filtration rate in the elderly, such as BIS1 (Berlin Initiative Study 1), have shown to be more precise and applicable than traditional ones (Cockroft-Gault, MDRD, CKD-EPI) [95,96,97,98,99], eGFR can still be misleading in this population due to reduced muscle mass [27]. Accordingly, therapeutic drug monitoring is essential in this population, especially when using narrow therapeutic index antibiotics [100,101,102]. A reduction in aminoglycoside use, recommended in the latest guidelines (no longer recommended in *Staphylococcus aureus* native-valve IE and shortened to 2 weeks in *Enterococcus faecalis* and streptococci with penicillin MIC > 0.125 μg/mL) [9], as well as aminoglycoside replacement by ceftriaxone in *E. faecalis* treatment [103,104,105,106], should be preferential in older people [107,108]. Despite recent efforts to improve vancomycin drug monitoring [100,101,109,110], daptomycin has shown a lower risk of clinical failure and treatment-limiting AEs than vancomycin [111,112,113,114] and should be preferentially used when available. Despite being a core recommendation, the benefit of adjunctive rifampicin therapy in staphylococcal prosthetic valve IE is uncertain [115,116]. It implies a high risk of clinically relevant drug interactions [117,118], which constitutes a differential problem in older polymedicated patients. In non-HACEK GNB etiology, combination therapy with quinolones may be associated with a better prognosis, but additional studies are needed to define the optimal treatment for these patients [51].

Additionally, prolonged intravenous antibiotic therapy in the elderly is troublesome due to poor peripheral venous access, the development of hyperactive delirium, a higher risk of catheter infection, and falls related to intravenous lines [94]. Furthermore, extended hospitalization stays for prolonged intravenous treatments have a considerable impact on functional status in older patients [76,119,120,121]. Outpatient parenteral antibiotic therapy (OPAT) has shown excellent results despite the use of broader selection criteria than those recommended by IDSA [10] in the recently published experience of the GAMES cohort [122]. However, OPAT at home, despite being a cost-effective alternative for older people [123], is not available in most settings, and age-related conditions also complicate attendance at day hospitals for this purpose. The partial oral treatment of endocarditis in clinically stable patients after initial intravenous antibiotic therapy has been shown to be non-inferior to continued intravenous antibiotic treatment in patients (including those ≥ 65 years old) with left heart endocarditis [124,125], also after a 5-year follow-up period [126]. Therefore, it could be an interesting approach to shorten the length of stay and limit the impact on the functional status of IE in the elderly. Additionally, treatment with long-acting lipoglycopeptides (administration once every 1–2 weeks) for consolidation treatment could also allow prompt hospital discharge [127] and avoid compliance problems of oral therapies in this population due to poor cognitive function, impaired hearing or vision, and complex regimens of polypharmacy [4]. 

## 8. Surgical Treatment: To Operate or Not to Operate?

Surgical treatment of IE continues to constitute a controversial debate both in academic and clinical scenarios, even more in elderly patients due to age-related higher surgical risk. Lack of consensus is based on the scarce and low-quality scientific evidence as well as on different tendencies among diverse groups and surgical scenarios.

The surgical indication is well-established based on cardiac and infectious complications that have not changed substantially in the latest international guidelines compared to previous ones. In both native and prosthetic valve IE, surgery is recommended in the presence of acute heart failure due to valve incompetence, major, or recurrent embolisms, large mobile vegetations, fever or persistent positive blood cultures despite adequate antibiotic therapy, new-onset conduction disturbances, abscesses, intracavitary fistulae, or aggressive microorganisms [9,10,128,129].

Nonetheless, despite several studies finding lower mortality in operated patients, older patients are less likely to be operated on [17,29,32,130,131]. ESC guidelines recommend assessing clinical status, comorbidities, and operative risk to guide the decision in patients with surgical indication [9]. The most frequently used risk scores in the surgical treatment of endocarditis (STS-IE, De Feo-Cotrufo, PALSUSE, Costa, RISK-E score, AEPEI score, EndoScore, SpecificEuroSCORE I and II, APORTE) include active IE as a highly weighted independent variable to predict mortality and adverse clinical events. However, no surgical score is currently validated for IE, and preoperative scores validated for other cardiac surgeries do not include geriatric parameters [4].

Even with similar demographics and surgical procedures, elderly patients are at higher risk of in-hospital mortality and complications after surgery for left-side IE than younger patients. Nevertheless, if such patients are stabilized by surgery and survive to discharge, mid-term outcomes are similar to those of the younger population [29,32,130,131].

Most authors conclude that surgical recommendations should be better implemented in the elderly to improve outcomes, but most studies lack a characterization of the geriatric profiles of these patients [94]. When the geriatric profile is assessed, it differs in operated patients from non-operated ones. Patients treated with surgery are usually younger, with fewer comorbidities and better functional and nutritional status [4,76]. Furthermore, although comorbidities such as renal dysfunction, cerebrovascular disease, previous valve surgery, and a poor clinical state are associated with a worse outcome in these patients, they do not influence the decision to operate as much as age and cardiac variables (especially left ventricular dysfunction), as the Euro Heart Survey showed [131].

Accurate surgical management planning and careful assessment before disease progression are mandatory to improve outcomes [132]. Regarding optimal timing, early surgery (i.e., interventions performed before finishing the whole antibiotic course for the responsible microorganism) to avoid complications is increasingly recommended [133], as it has the potential to improve survival also in elderly patients [71]. The most frequent factors leading to shortened time till surgery are the presence of prosthetic valve IE or highly aggressive microorganisms (usually staphylococci, Gram-negative bacilli, and, less frequently, fungi) responsible for the severe and fast worsening of the patient clinical status [9,129,134].

Till better prognostic scores for surgical treatment of IE, including frailty and geriatric items, are developed, “Endocarditis teams” formed by geriatricians, cardiologists, cardiac surgeons, and infectious disease specialists should assess each patient to decide on the best management of individual base [18]. In high surgical risk patients, the transcatheter fixing of left-sided valve regurgitation could help to reduce the high 1-year mortality attributed to severe infection-related valve damage [27,135,136]. Lower rates of surgical complications and early patient mobilization with these techniques are particularly important in this population [131].

## 9. IE after TAVI: A Frequent Specific Situation in the Elderly Patient

Due to the relatively low procedural risks of TAVI compared to surgical aortic valve replacement, it is increasingly used, particularly in older patients. The incidence of IE after TAVI is comparable with the one after surgical aortic valve replacement and is around 1% per person-year [137,138,139]. However, the morbidity and mortality associated with IE after TAVI are higher, probably due to a worse clinical profile of the patients who usually are older, more fragile, and present multiple comorbidities. In this sense, in-hospital and one-year mortality rates in IE after TAVI have been reported to be as high as 30–40% and up to 66%, respectively [137,138,139].

The main risk factors for IE after TAVI are mainly related to the patients’ baseline clinical status, especially heart failure, cardiovascular risk factors, or significant aortic regurgitation concomitant to aortic stenosis [137]. Still, many events that can occur during the implantation process are also implicated in the incidence of IE, such as orotracheal intubation, need for permanent pacemaker placement, cardiac arrest, or major bleeding [140]. Furthermore, recent research has reported a higher risk of IE in patients undergoing implantation of self-expandable prostheses [139].

Although the etiology of IE after TAVI varies among different registries, it is predominantly caused by *Enterococcus* spp. in 25–30%, of cases, specifically in early infections within the first year of implantation, followed by *Staphylococcus* and *Streptococcus* spp. [139,140,141].

The diagnosis of IE after TAVI is complex because frequently, these patients present atypical symptoms and echocardiographic findings. Fever is the most prevalent symptom, but it is less frequent than in cases of IE in the general population [22]. In addition, a new regurgitant heart murmur, an important sign for the diagnosis of IE, may be less valuable in TAVI patients because of their high rate of residual paravalvular leaks and valvular regurgitation after the procedure [142]. Vegetations are commonly detected, but periannular complications are found in up to 50% of the cases [141].

The extremely high surgical risk of the patients with TAVI and the technical difficulties of an explantation of a large stent in ascending aorta are probably the main reasons that discourage surgical intervention in patients with IE after TAVI [139]. Nevertheless, patients with complications derived from IE such as heart failure who do not undergo surgery present a mortality rate of around 90% [141]. An individualized therapeutical approach considering the patient’s functional status and frailty and new options such as valve-in-valve techniques are promising in this complex disease [139].

## 10. Conclusions

IE epidemiology has changed from a disease mainly related to young people and caused by *Streptococcus viridans* to one generally associated with older patients, enterococcal etiology, and mostly linked to health care. Improving our knowledge of the specificity of IE in the elderly is essential, since recent guidelines on IE do not provide special considerations for its management, and the extent to which the recommendations can be applied to these patients remains poorly evaluated due to the lack of large multicentric cohorts and the heterogeneity of this population. We need more clinical trials in this group to assess the impact of multidisciplinary and individualized approaches on mortality and quality of life, clinical decision-making, and cost outcomes. Nonetheless, cooperative multidisciplinary management is key to performing a more global patient assessment and deciding the best therapeutic approach for elderly patients with IE.

## Figures and Tables

**Table 1:** Special characteristics of the epidemiology of EI in the elderly in age-focused studies.

	Most Frequent Comorbidities (%)	Nosocomial Rate (%)	Valvular Prosthesis (%)	Endovascular Device (%)	Mortality In-Hospital (%)	Mortality at 1 Year (%)
Durante-Magoni et al. (2008) [32] *n* = 2759 (>70 y, *n* = 773; <70 y, *n* = 1985)	Chronic illness (54.1) * Diabetes mellitus (22.4) * Cancer (14.9) *	20.3 *	26.5 *	ND	25.8 *	ND
López-Wolf et al. (2011) [31] *n* = 618 (>79 y, *n* = 34; <79 y, *n* = 584)	Chronic anemia (30.3) * Diabetes mellitus (24.2) * Immunodepression (12.1) *	ND	23.5 *	11.3*	20.6 *	ND
Bassetti et al. (2014) [16] *n* = 436 (>75 y, *n* = 137; <75 y, *n* = 299)	Chronic heart failure (47.7) * Chronic renal failure (29.2) * Cancer (25) *	27.7 *	40.2 *	ND	22.6	ND
Oliver et al. (2017) [30] *n* = 454 (>80 y, *n* = 51; <80 y, *n* = 403)	High blood pressure (58.8) * History of cancer (29.4) * CKD (27.5) *	23.5 *	41.2 *	4	15.7	37.3 *
Armiñanzas et al. (2019) [12] *n* = 3120 (>80 y, *n* = 502; <80 y, *n* = 2618)	Congestive heart failure (40.5) * Diabetes mellitus (30.7) * Coronary arterial disease (25.7) *	30.7	26.3 *	16.3 *	34.7 *	20.4 *
Menchi-Elanzi et al. (2020) [17] *n* = 72 (>80 y, *n* = 18; <80 y, *n* = 54)	Heart disease (72.2) * Diabetes mellitus (27.8) History of cancer (5.6)	ND	38.9 *	16.7	5.6	ND
Kiriyama et al. (2021) [33] *n* = 20,667 (>80 y, *n* = 4990; <80 y, *n* = 15,677)	High blood pressure (31.8) * Diabetes mellitus (15.9) * Atrial Fibrillation (14) *	ND	0.7 *	ND	22.8 *	ND

* This data showed a statistically significant difference compared with other age groups. ND: no data.

**Table 2:** Most frequent clinical manifestations in IE in the general population [29,30,50].

Clinical Presentation of IE	
Signs	Symptoms
New-onset heart murmur (50–85%)	Fever (90%)
Congestive heart failure (30%)	Chills
New conduction disturbances (2%)	Malaise
Disturbances in CNS (stroke, meningitis…) (14%)	Dyspnea
Peripheral septic abscesses or emboli	Anorexia
(renal, splenic, vertebral…) (5%)	Weight loss
Septic pulmonary emboli (6%)	Generalized weakness
Fever or sepsis of unknown origin	Back pain
Splinter hemorrhages (8%)	
Roth’s spots (8%)	
Acute kidney failure (23%)	
Anemia (Hb <10 g/dL) (20%)	

**Table 3:** Tools for global assessment of the elderly patient.

Domain	Measure	Range of Scores
Frailty	Clinical Frailty Scale (CFS) [82]	1–9
	Fried frailty criteria [77]	0–5
Basic activities of daily living (ADL)	Barthel Index for Activities of Daily Living (ADL) [83]	0–100 *
	Katz Index [84]	0–6 *
Instrumental ADL (IADL)	Lawton IADL [85]	0–7 *
Mobility	Timed Up and Go [86]	0–30 sec
	Tinetti Assessment Tool [87]	0–28
Cognition	Mini-Mental State Examination (MMSE) [88]	0–30 *
	Confusion Assessment Method [89]	0–4
Depression	Geriatric Depression Scale [90]	0–30
Co-morbidity	Age-adjusted Charlson Co-morbidity Index [91]	0–≥ 5
Nutrition	Mini Nutritional Assessment [92]	0–30 *

* Indicate higher score = better functional status. Unmarked ranges indicate higher score = worse functional status.

## Data Availability

Not applicable.
